# The human gallbladder microbiome is related to the physiological state and the biliary metabolic profile

**DOI:** 10.1186/s40168-019-0712-8

**Published:** 2019-07-04

**Authors:** Natalia Molinero, Lorena Ruiz, Christian Milani, Isabel Gutiérrez-Díaz, Borja Sánchez, Marta Mangifesta, José Segura, Isabel Cambero, Ana Belén Campelo, Carmen María García-Bernardo, Ana Cabrera, José Ignacio Rodríguez, Sonia González, Juan Miguel Rodríguez, Marco Ventura, Susana Delgado, Abelardo Margolles

**Affiliations:** 1 Department of Microbiology and Biochemistry, Dairy Research Institute of Asturias, Spanish National Research Council (IPLA-CSIC), Paseo Río Linares s/n, 33300 Villaviciosa, Asturias Spain; 2 Departmental sections of Food Technology, and Nutrition and Food Science, Complutense University of Madrid, Madrid, Spain; 3 Laboratory of Probiogenomics, Department of Chemistry, Life Sciences, and Environmental Sustainability, University of Parma, Parma, Italy; 4 Area of Physiology, Department of Functional Biology, University of Oviedo, Asturias, Spain; 5 General Surgery Service, Central University Hospital of Asturias, Asturias, Spain; 6 General and Digestive Surgery Service, Cabueñes Gijon University Hospital, Asturias, Spain; 7 Microbiome Research Hub, University of Parma, Parma, Italy

**Keywords:** Bile microbiota, Cholelithiasis, Microbial bile metabolites, Gallstones patients

## Abstract

**Background:**

The microbial populations of the human intestinal tract and their relationship to specific diseases have been extensively studied during the last decade. However, the characterization of the human bile microbiota as a whole has been hampered by difficulties in accessing biological samples and the lack of adequate methodologies to assess molecular studies. Although a few reports have described the biliary microbiota in some hepatobiliary diseases, the bile microbiota of healthy individuals has not been described. With this in mind, the goal of the present study was to generate fundamental knowledge on the composition and activity of the human bile microbiota, as well as establishing its potential relationship with human bile-related disorders.

**Results:**

Human bile samples from the gallbladder of individuals from a control group, without any record of hepatobiliary disorder, were obtained from liver donors during liver transplantation surgery. A bile DNA extraction method was optimized together with a quantitative PCR (qPCR) assay for determining the bacterial load. This allows the selection of samples to perform functional metagenomic analysis. Bile samples from the gallbladder of individuals suffering from lithiasis were collected during gallbladder resection and the microbial profiles assessed, using a 16S rRNA gene-based sequencing analysis, and compared with those of the control group. Additionally, the metabolic profile of the samples was analyzed by nuclear magnetic resonance (NMR). We detected, for the first time, bacterial communities in gallbladder samples of individuals without any hepatobiliary pathology. In the biliary microecosystem, the main bacterial phyla were represented by *Firmicutes*, *Bacteroidetes*, *Actinobacteria*, and *Proteobacteria*. Significant differences in the relative abundance of different taxa of both groups were found. Sequences belonging to the family *Propionibacteriaceae* were more abundant in bile samples from control subjects; meanwhile, in patients with cholelithiasis members of the families *Bacteroidaceae*, *Prevotellaceae*, *Porphyromonadaceae*, and *Veillonellaceae* were more frequently detected. Furthermore, the metabolomics analysis showed that the two study groups have different metabolic profiles.

**Conclusions:**

Our results indicate that the gallbladder of human individuals, without diagnosed hepatobiliary pathology, harbors a microbial ecosystem that is described for the first time in this study. Its bacterial representatives and metabolites are different from those detected in people suffering from cholelithiasis. In this regard, since liver donors have been subjected to the specific conditions of the hospital’s intensive care unit, including an antibiotic treatment, we must be cautious in stating that their bile samples contain a physiologically normal biliary microbiome. In any case, our results open up new possibilities to discover bacterial functions in a microbial ecosystem that has not previously been explored.

**Electronic supplementary material:**

The online version of this article (10.1186/s40168-019-0712-8) contains supplementary material, which is available to authorized users.

## Background

Bile is a biological fluid, mainly constituted by bile acids (BA), cholesterol, phospholipids, and proteins. Bile is synthesized in the liver and stored in the gallbladder. Its main physiological function is to facilitate fat absorption in the small intestine during digestion [1]. Several bile-related disorders can modify bile functionality, but the most frequent is the generation of gallstones, either in the gallbladder or in the bile duct, the so-called cholelithiasis, with a prevalence among adults normally above 10% [2]. Gallstone formation is attributed to a combination of environmental and genetic causes, typically linked to cholesterol supersaturation. The primary constituent of gallbladder stones is cholesterol, whereas calcium bilirubinate predominates in pigmented bile duct stones. To date, cholecystectomy remains the most effective treatment option for chronic carriers with gallbladder lithiasis [3].

The landscape of microorganisms inhabiting our gastrointestinal tract has been extensively studied during the last few years [4–6]. Furthermore, we currently know that some biological fluids in different locations in our body also have an autochthonous microbiota that suffers alterations depending on the physiological state of the host [7, 8]. However, very little is known about the microbial inhabitants of human bile. Current knowledge is mainly limited to a few species of cultivable bacteria that have been associated with physiological disorders such as cholelithiasis. In this regard, our understanding of the exact contribution of bacteria in gallstone formation is very limited, although a possible association between bacteria and the etiology of gallstones has been suggested and enterobacteria are frequently isolated from bile aspirates or gallbladder bile from cholelithiasis patients [9–12]. Only very recently, a few authors have analyzed, using culture-independent techniques, the microbiota of the biliary tract and the gallbladder and its association with bile-related diseases. In these studies, it was shown that *Enterobacteriaceae* members are abundant microorganisms in the biliary tract of acute cholecystitis and gallstone patients [13, 14]. It was also observed that oral cavity and respiratory tract microorganisms were more prevalent than intestinal microorganisms in the microbiota of the common bile duct of gallstone disease patients [15] or that the genera *Prevotella*, *Streptococcus*, *Veillonella*, *Fusobacterium*, and *Haemophilus* are prevalent in the bile microbial communities of the bile duct of primary sclerosing cholangitis patients [16], among other findings [13, 17, 18]. All these works demonstrated that a microbial community is present in the human gallbladder and the bile duct in some hepatobiliary disorders. Nevertheless, the human bile microbiota in individuals without any bile or liver-related disorder had not been explored until now.

In a previous work, we characterized the gallbladder microbiota of healthy pigs [19]. This fact, together with our experience in bacterial bile resistance mechanisms, led us to believe that the human gallbladder may contain an autochthonous microbiota, and the ability of resident bacteria to live in, survive, and colonize bile must necessarily reflect a specific bacterial physiology, well adapted to face the environmental challenges found in such a specific niche, and thus different from the rest of the microorganisms inhabiting other gastrointestinal habitats. Thus, we performed the first study focused on the characterization of human gallbladder microbiota in a group of individuals free from hepatobiliary diseases (control group) vs a group with diagnosed cholelithiasis (non-lithiasis vs lithiasis), with the aim of generating new knowledge about bile microbial profiles, functions, and activities and to contribute to complete our understanding of the complex landscape of different microbiotas in the human body (Table 1).

**Table 1:** Demographic and clinical features of cholelithiasis patients

Subject	Age (years)	Sex	Clinical history	Altered parameters in blood test (with higher levels than those considered normal)^a^
C-01	73	F	Non-cirrhotic portal hypertension. Alteration of liver function due to chemotherapy. Digestive hemorrhage (2 years before collecting the bile sample)	ALP, AST, and GGT
C-02	67	M	Hemicolectomy (3 years before collecting the bile sample)	GGT, TB, DB, and TG
C-03	60	F	Hypercholesterolemia	–
C-04	58	F	–	AST and GGT
C-05	67	M	–	DB and TB
C-06	55	F	Obesity type II (BMI 38.5)	ALT, AST, Glu, GGT, and TC
C-07	27	F	–	–
C-08	50	M	Obesity type I (BMI 32.2) Acute lithiasic pancreatitis (7 months before collecting the bile sample)	GGT, LDL, TC, and TG
C-09	38	F	–	LDL and TC
C-10	33	F	–	–
C-11	36	F	Obesity type II (BMI 36.1)	ALT and GGT
C-12	44	F	–	TG
C-13	50	F	–	ALT and AST
C-14	70	F	–	DB, GGT, and TB

– no relevant information, *BMI* body mass index, *ALP* alkaline phosphatase, *ALT* alanine aminotransferase, *AST* aspartate aminotransferase, *DB* direct bilirubin, *GGT* gamma-glutamyl transferase, *Glu* glucose, *LDL* cholesterol LDL, *TB* total bilirubin, *TC* total cholesterol, *TG* triglycerides, *F* female, *M* male

^a^At the time of sampling

## Results

### Bacterial load in bile samples

**Fig. 1:**
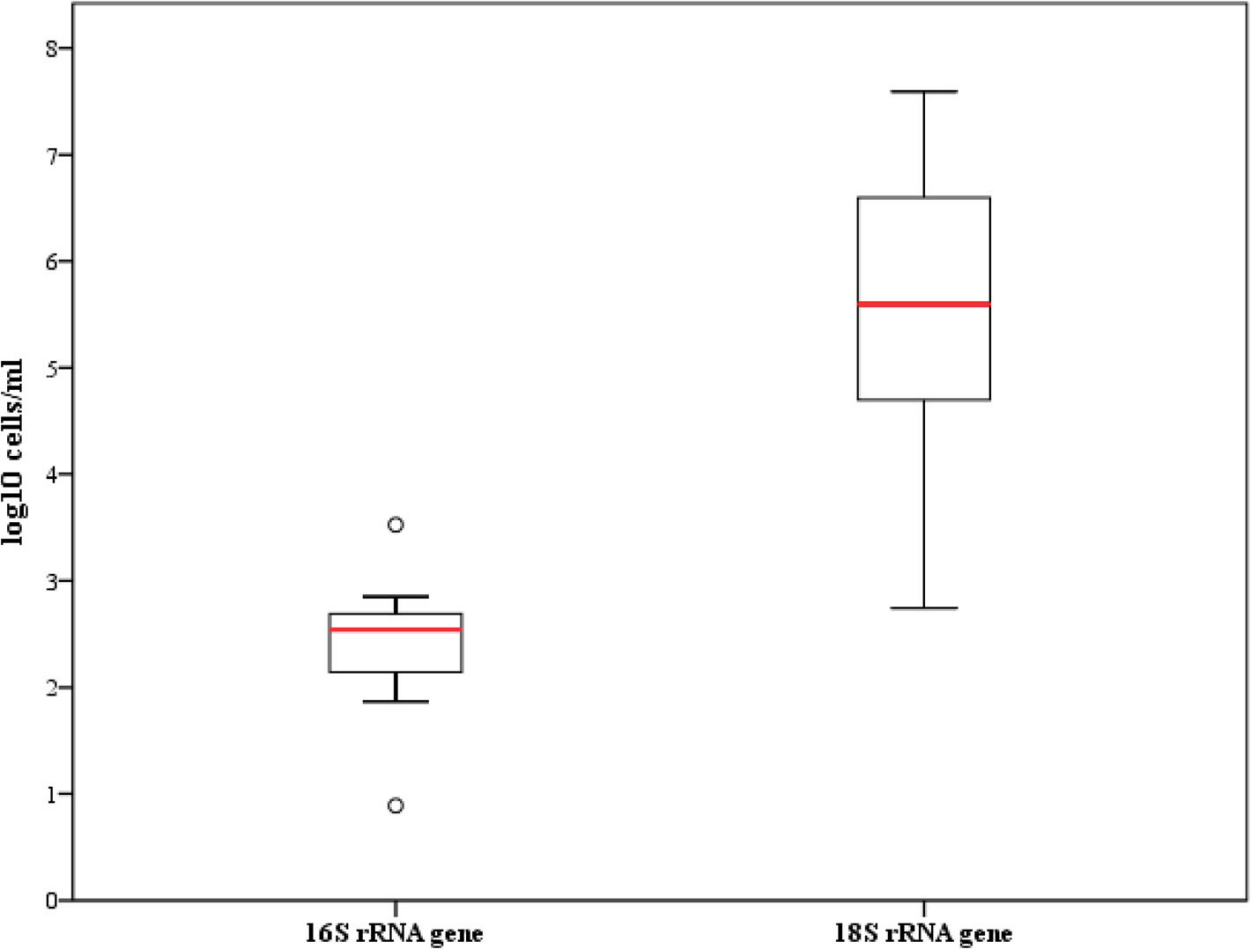
Boxplots representing 16S rRNA gene and 18S rRNA gene levels in the control group (*n* = 13). The central rectangles represent interquartile ranges (IQR), the lines inside the rectangles show the median, and the whiskers indicate the maximum and minimum values. The dots outside the rectangles are suspected outliers (> 1.5 × IQR). Statistically significant differences (*p* value < 0.05) between the two variables (16S rRNA gene and 18S rRNA gene) were found (Mann-Whitney *U* test)

In order to perform further molecular analyses, we firstly optimized a method for DNA extraction and quantification from human bile. For this purpose, bile samples from liver donors that did not meet the inclusion criteria for investigating the microbiome (see the “Methods” section for details) were used to extract biliary DNA following a phenol-based protocol. A quantitative PCR (qPCR) assay was developed to determine the bacterial load. General primer pairs, targeting the 16S rRNA gene of prokaryotic microorganisms and the18S rRNA gene of eukaryotic cells, were used. DNA quantification and recovery after artificial enrichment of these bile samples with different amounts of bacterial cells was performed. Total DNA was extracted and used as a template for qPCR validation experiments, and the threshold cycle (Ct) values were used to calculate the efficiency and the limit of quantification. Dilution series of eukaryotic and prokaryotic DNA, obtained from cell cultures of *Lactococcus lactis* NZ9000 and HT29 cells, allowed us to perform a linear regression analysis based on the Ct data, yielding a high coefficient of determination (*R*^2^ > 0.99). The lowest concentration of the standard was close to the Ct value of the non-template control, so 10^2^ bacterial cells/ml was established as the limit of detection of our assay. Afterwards, DNA samples obtained from the bile of a group of 13 liver donors (see the “Methods” section for general characteristic of the selected control group of donors) were analyzed with the qPCR assay and the bacterial load and the ratio of prokaryotic DNA with respect to eukaryotic DNA established. We observed that bacterial cell counts between individuals did not vary substantially (Fig. 1). The proportion of eukaryotic DNA was superior by at least tenfold compared to prokaryotic DNA in all cases.

Overall, the qPCR assay was useful to determine the levels of total bacteria in bile and allowed us to select samples for performing shotgun sequencing.

### Functional metagenomic analysis of bile samples

Three bile samples (encoded in this study as H-04, H-05, and H-06) were selected based on the results obtained by qPCR (16S rRNA gene/18S rRNA gene ratio ≤ 0.01) and DNA concentration and quality (≥ 15 ng/μl and a ratio 260/280 ≥ 1.6). The metagenomic analysis performed by shotgun sequencing resulted in a mean of 2,556,103 raw sequences per sample obtained, of which a total of 7,169,256 proved to be of high quality considering the three samples together. After the removal of human DNA, the numbers of final reads were 1,963,038 for H-04, 342,649 for H-05, and 80,384 for H-06. These sets of sequences served as input for further analyses. Taxonomy assignment based on coding reads against non-redundant NCBI database with BLASTx and the metagenome analyzer MEGAN5 revealed that most of the quality-filtered reads resulted from bacteria, although the presence of archaea, virus, and other eukaryotic microorganisms was also noticed (Table 2). Within the bacteria domain, four main phyla, *Firmicutes*, *Bacteroidetes*, *Actinobacteria*, and *Proteobacteria*, predominate in the bile microbiome. Other phyla, such as *Verrucomicrobia*, *Chlamydiae*, *Acidobacteria*, *Planctomycetes*, *Cyanobacteria*, *Spirochaetes*, and *Fusobacteria*, were noticed in the three samples at low percentages (mean between 0.05 and 0.5%) after normalization against the total number of sequences assigned to bacteria.

**Table 2:** Percentages (%) of quality and human filtered reads assigned at the ranks of superkingdom and phylum from three control bile samples subjected to shotgun metagenomics

Taxonomic assignment		Sample code		
Superkingdom	Phylum^a^	H-04	H-05	H-06
Not assigned		32.67	20.96	33.13
Archaea		0.02	0.06	0.04
Eukaryota		2.11	0.21	1.83
Viruses		0.12	5.58	0.20
Bacteria		65.08	73.19	64.79
	*Actinobacteria*	13.17	2.91	12.23
	*Bacteroidetes*	34.36	34.22	35.60
	*Firmicutes*	22.47	56.51	23.10
	*Proteobacteria*	27.80	5.46	27.06
	*Verrucomicrobia*	0.16	0.43	0.25
	*Chlamydiae*	0.73	0.01	0.55
	*Acidobacteria*	0.18	0.04	0.14
	*Planctomycetes*	0.20	0.05	0.20
	*Spirochaetes*	0.14	0.07	0.12
	*Fusobacteria*	0.09	0.05	0.10
	*Tenericutes*	0.05	0.08	0.09
	*Deinococus-Thermus*	0.02	0.00	0.01
	*Fibrobacteres*	0.00	0.00	0.02
	*Synergistetes*	0.00	0.02	0.00
	*Cyanobacteria*	0.11	0.03	0.08
	*Chloroflexi*	0.03	0.01	0.03
	*Chlorobi*	0.00	0.00	0.02
	*Nitrospirae*	0.00	0.00	0.01
	Unclassified bacteria	0.49	0.21	0.38

^a^Abundances of different phyla are related to total assigned bacteria sequences

**Fig. 2:**
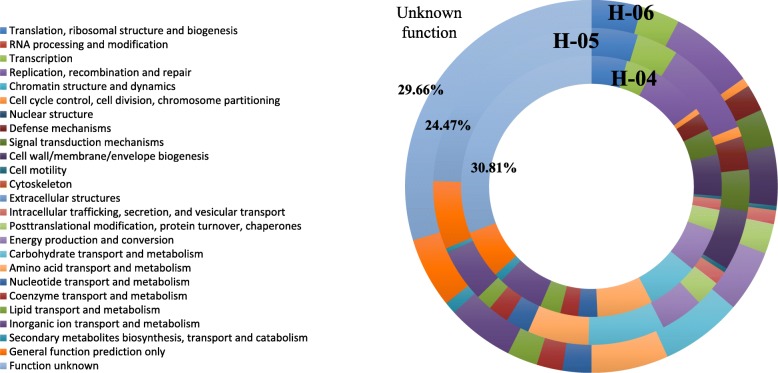
Distribution of Clusters of Orthologous Groups (COGs) in three bile samples from control group (H-04, H-05, and H-06). The results show the percentage of sequences assigned to different metabolic functions (relative to all sequenced microbes). Secondary metabolite biosynthesis includes antibiotics, pigments, and non-ribosomal peptides. Inorganic ion transport and metabolism includes phosphate, sulfate, and various cation transporters

When performing the gene annotation and functional classification of the metagenomes obtained for the three samples, assignation results for the main functional categories of COG (Clusters of Orthologous Groups) were closely related. The COG functional classes, as observed in Fig. 2, were also similar to those found in the intestinal microbiome, although between 24 and 31% of the sequences were annotated as “unknown function.” The search for activities related to the metabolism of cholesterol and bile salts in the MetaCyc metabolic pathway database showed differences between the three samples. As shown in Table 3, a clear difference was observed for the superpathway of cholesterol biosynthesis and to a minor extent glycocholate metabolism, with 2 to 4 times more genes related with this pathway in samples H-04 and H-05, respectively, as compared to H-06. Additionally, when comparing with similar activities against metagenomes from fecal samples of five healthy individuals (see the “Methods” section for accession numbers and reference), differences were observed for cholesterol oxidase genes, more abundant in bile. On the contrary, bacterial genes for glycocholate metabolism were more abundant in the fecal datasets.

**Table 3:** Microbial functional genes related to the metabolism of cholesterol and BA assigned (ratio 1/10,000 reads) through MetaCyc metabolic pathway database in three metagenomes from human bile and five fecal metagenomes from healthy subjects from a previous study [20]

	Bile samples			Fecal samples					Average bile	Average gut
Pathway	H-04	H-05	H-06	2HS	26HS	30HS	31HS	32HS		
Cholesterol biosynthesis I	0.13	0.02	0.29	0.00	0.00	0.00	0.08	0.00	0.15 ± 0.11	0.02 ± 0.03
Cholesterol biosynthesis II (via 24,25-dihydrolanosterol)	0.13	0.02	0.29	0.00	0.00	0.00	0.08	0.00	0.15 ± 0.11	0.02 ± 0.03
Cholesterol biosynthesis III (via desmosterol)	0.13	0.02	0.29	0.00	0.00	0.00	0.08	0.00	0.15 ± 0.11	0.02 ± 0.03
Superpathway of cholesterol biosynthesis	0.37	3.29	15.31	0.12	0.21	0.25	1.06	0.25	6.32 ± 6.47	0.38 ± 0.34
Superpathway of cholesterol degradation I (cholesterol oxidase)	1.06	0.17	0.21	0.41	0.46	0.39	0.48	0.71	0.48 ± 0.41	0.49 ± 0.11
Superpathway of cholesterol degradation II (cholesterol dehydrogenase)	1.52	0.34	0.36	0.54	0.5	0.47	0.62	0.81	0.74 ± 0.55	0.59 ± 0.12
Cholesterol degradation to androstenedione I (cholesterol oxidase)	0.35	0.02	0.07	0.00	0	0.00	0.01	0.02	0.15 ± 0.15	0.01 ± 0.01
Cholesterol degradation to androstenedione II (cholesterol dehydrogenase)	0.74	0.05	0.21	0.00	0.00	0.00	0.06	0.02	0.33 ± 0.29	0.02 ± 0.02
Bile acid biosynthesis, neutral pathway	0.61	0.07	0.29	0.06	0.06	0.02	0.11	0.05	0.32 ± 0.22	0.06 ± 0.03
Glycocholate metabolism (bacteria)	1.58	2.71	0.72	10.58	15.03	12.4	15.53	12.35	1.67 ± 0.81	13.18 ± 1.84
Cholate degradation (bacteria, anaerobic)	0.35	0.46	0.14	0.97	0.77	0.41	0.62	0.38	0.32 ± 0.13	0.63 ± 0.22

### Bile microbiota load and compositional profiles: comparison between lithiasis and non-lithiasis

**Fig. 3:**
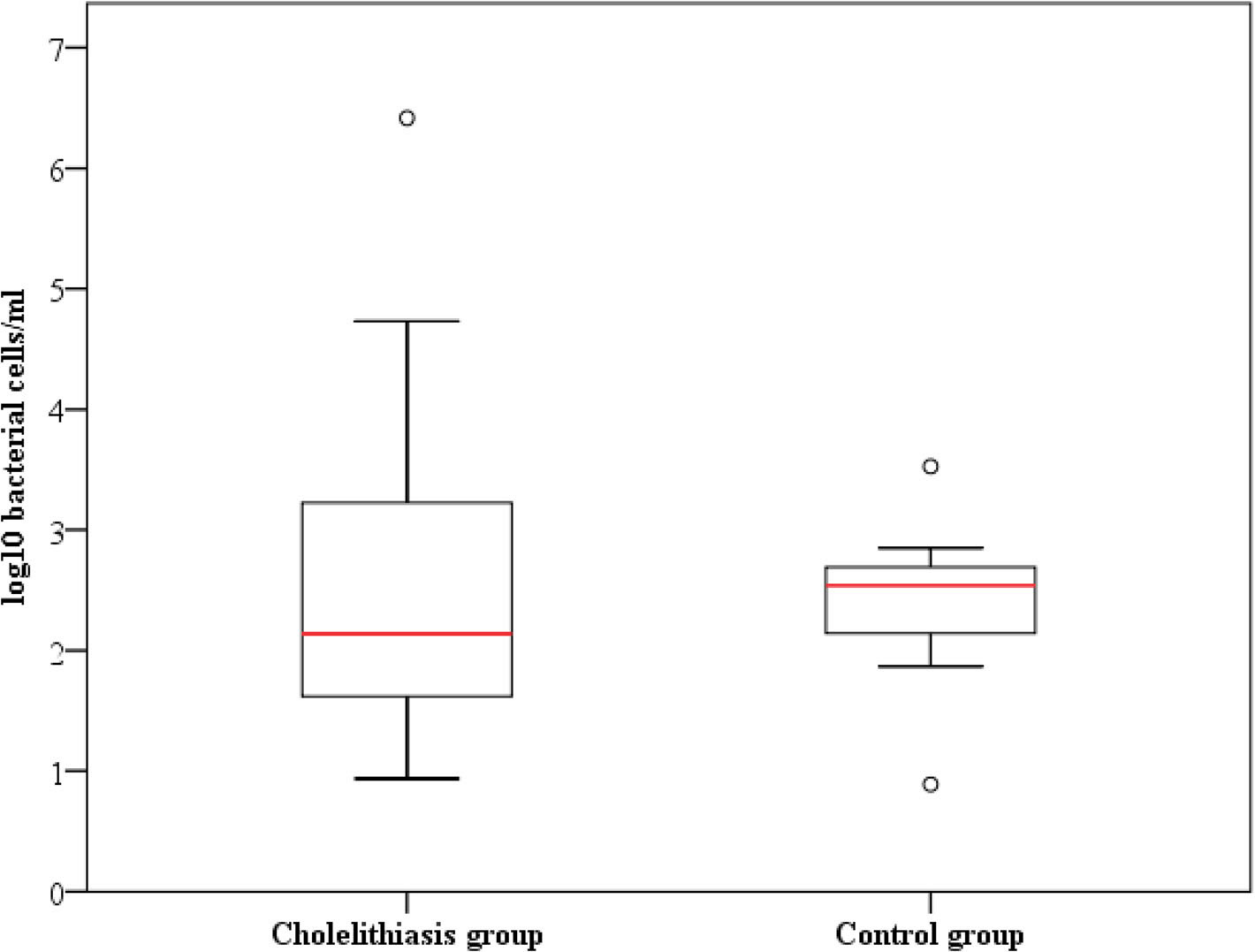
Boxplots representing 16S rRNA gene levels between cholelithiasis (*n* = 14) and control group (*n* = 13). The central rectangles represent interquartile ranges (IQR), the lines inside the rectangles show the median, and the whiskers indicate the maximum and minimum values. The dots outside the rectangles are suspected outliers (> 1.5 × IQR). Statistically significant differences (*p* value < 0.05) between the two groups of the study were not found (Mann-Whitney *U* test)

To compare the human gallbladder microbiota in individuals with no diagnosed hepatobiliary diseases vs a disease state, bile samples were obtained during surgery from patients (*n* = 14) diagnosed with cholelithiasis. The bacterial load was determined by qPCR, as for the control group (*n* = 13), and the results are shown in Fig. 3. No statistical differences were observed between either group for the total number of bacterial cells (Mann-Whitney *U* test, *p* value < 0.05), but much more variation (reaching levels of 10^4^–10^6^ in some cases) was found in the bile of patients with gallstones.

Bile microbial composition was determined by high-throughput sequencing by paired-end Illumina technology of 16S rRNA gene amplicons. In order to evaluate the potential DNA contamination associated to low bacterial biomass samples, three “blank” controls with no template DNA (adding ultrapure molecular biology grade water instead of bile for the DNA extraction protocol) were also processed for sequencing. Bacterial profiles and diversity from bile of both groups were compared. On average, a total of 74,885 raw reads per sample were obtained in the control group, and 100,510 in the cholelithiasis group. After quality and chimera filtering, a mean of 63,473 and 41,997 high-quality partial 16S rRNA gene sequences were retrieved, respectively. Sequences were classified, using QIIME and SILVA database. In accordance with the shotgun metagenome results, the main phyla found in bile were *Firmicutes*, *Bacteroidetes*, *Actinobacteria*, and *Proteobacteria*. Significant differences in the relative abundance of different taxa present in the bile of both groups were found after the application of the Metastats statistical method with a false discovery rate (FDR correction), adjusted following the Benjamini-Hochberg method to 0.25. At phylum level, *Bacteroidetes* was statistically less abundant in the bile of control subjects (13.49% with respect to 24.00% in cholelithiasis). Sequences belonging to the family *Propionibacteriaceae* were more abundant in bile samples from the control group (mean relative abundance 10.77%) compared with samples from patients with stones in the gallbladder (mean relative abundance 0.59%); meanwhile in patients, members of the families *Bacteroidaceae*, *Prevotellaceae*, *Porphyromonadaceae*, and *Veillonellaceae* were more frequently detected (10.21%, 3.23%, 2.36%, and 1.78%, respectively) (Table 4). Classification was assigned to the genus level when possible; otherwise, the closest taxonomic rank was given, preceded by unknown member “U. m.” (Table 5). In cholelithiasis patients, within the *Bacteroidaceae* family, *Bacteroides* was the genus that showed a significantly higher proportion, as compared with the control group (mean 10.21% vs 2.74% of relative abundance, respectively, *p* value = 0.001). Within the family *Veillonellaceae*, the genus *Dialister* also showed a significantly higher representation in the group of patients with gallstones. Moreover, a significantly higher percentage of assigned reads to the enterobacteria *Escherichia-Shigella* was observed in the bile of these patients. However, sequences assigned to other genera from the Alpha (*Bradyrhizobium*, *Methylobacterium*, and *Sphingomonas*) and Gamma (*Acidibacter* and *Brevundimonas*) divisions of the *Proteobacteria* phylum were more abundant in the control group. In contrast, no amplification was observed from any of the three blank controls included in this study. In this regard, to further support the existence of an autochthonous biliary microbiota, different cultivation techniques were used. We were able to identify several isolates from a selection of bile samples, and in all cases, the microbial profile determined by 16S rRNA gene sequencing contained sequences (at genus or family level) that matched with the corresponding isolates of each sample (data not shown)

**Table 4:**
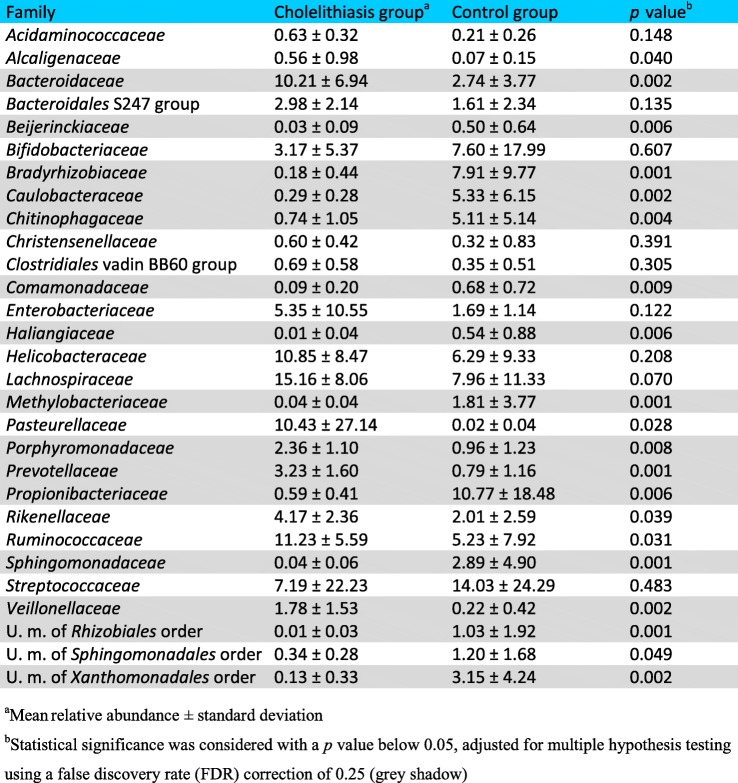
Differences in microbial relative abundance (% of sequences) in bile at family level between cholelithiasis patients and the control group. Only families with a mean relative abundance higher than 0.5% are presented. Only families that were detected in more than half of the samples in each group were considered for the analysis

**Table 5:**
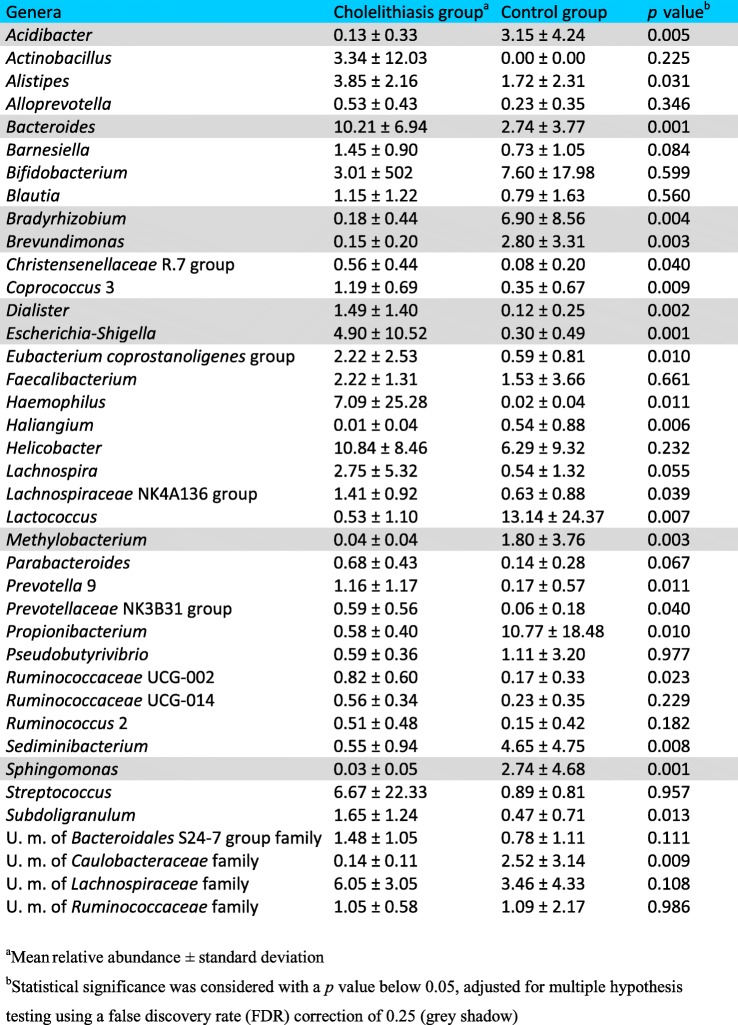
Differences in microbial relative abundance (% of sequences) in bile at genus level between cholelithiasis patients and the control group. Only genera with a mean relative abundance higher than 0.5% are presented. Only genera that were detected in more than half of the samples in each group were considered for the analysis

**Fig. 4:**
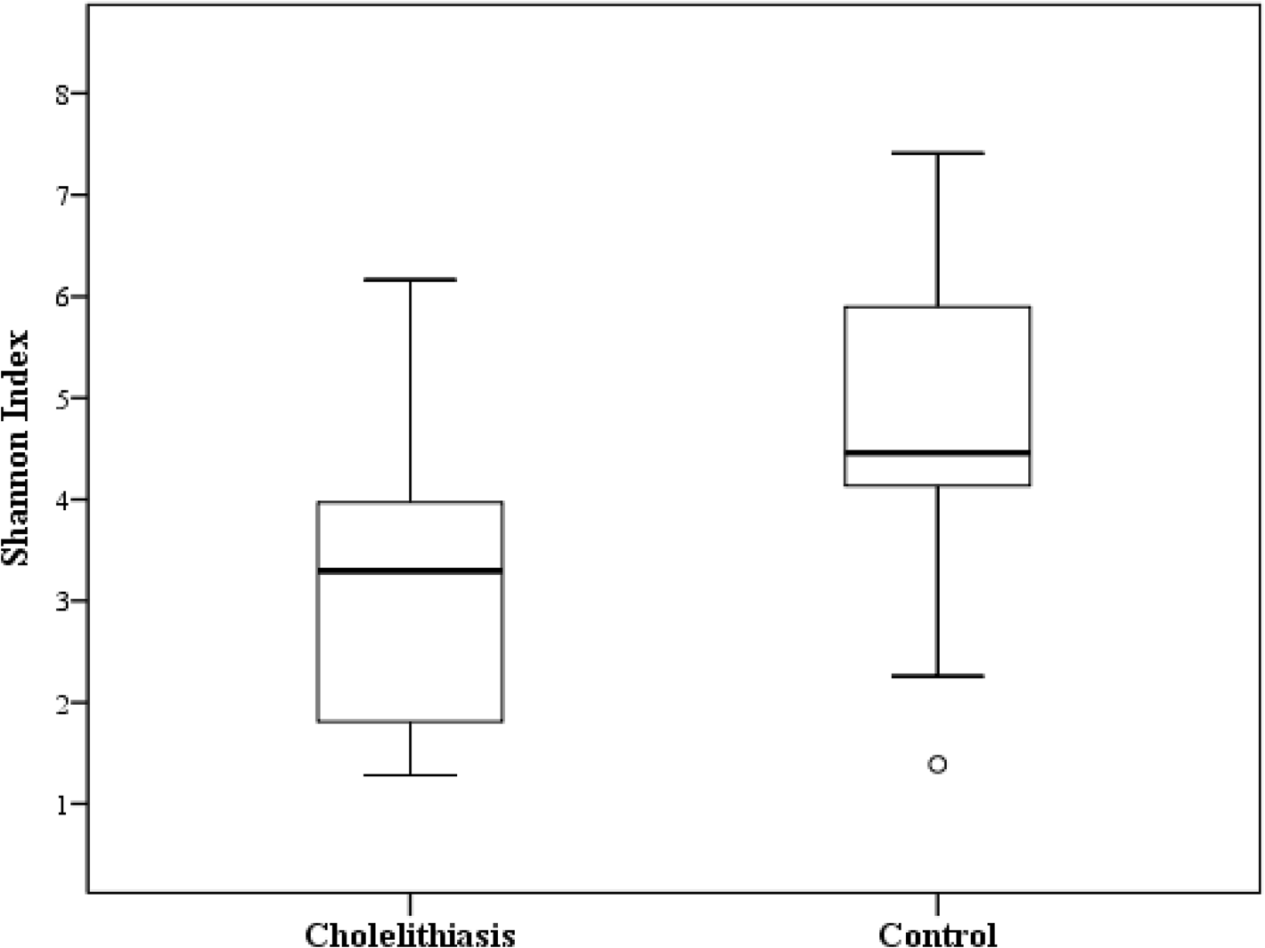
Comparison of Shannon’s diversity indices in cholelithiasis (*n* = 14) and control (*n* = 13) groups. The central rectangles represent interquartile ranges (IQR), the lines inside the rectangles show the median, and the whiskers indicate the maximum and minimum values. The dots outside the rectangles are suspected outliers (> 1.5 × IQR). Statistically significant differences (*p* value < 0.05) between groups were found (Mann-Whitney *U* test)

**Fig. 5:**
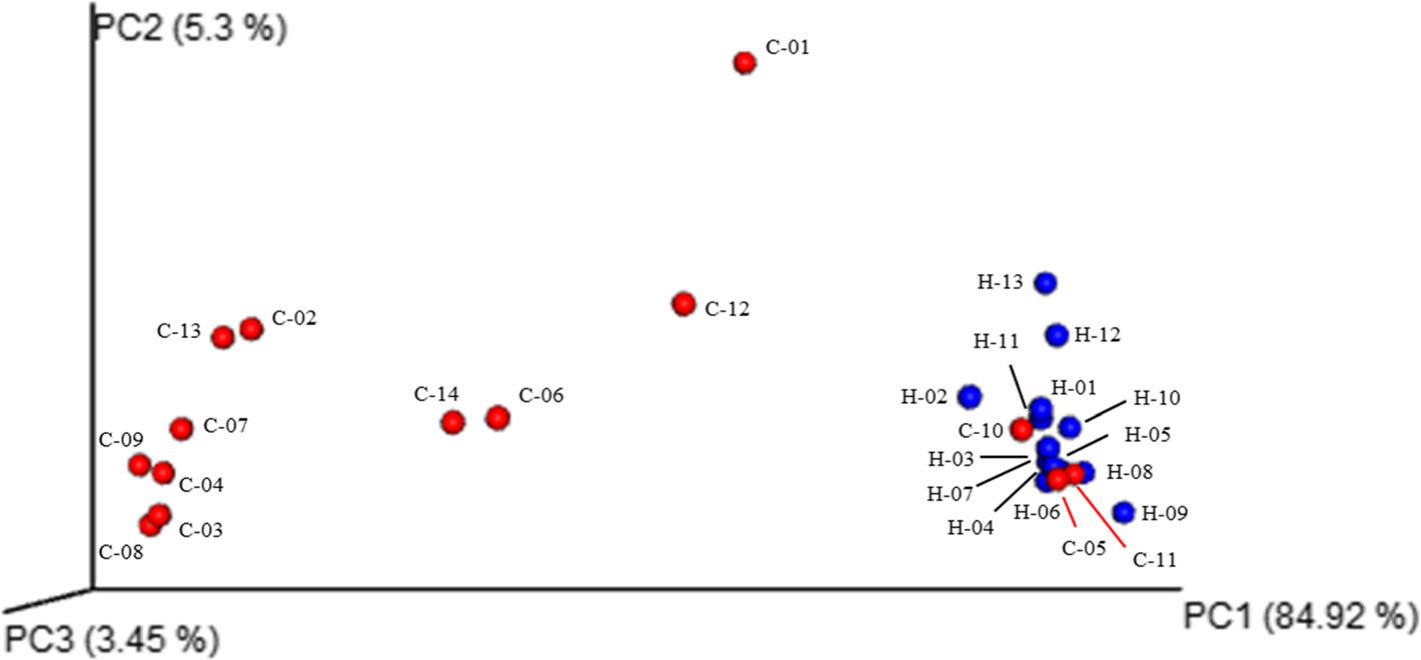
Principal Coordinate Analysis (PCoA) plot of weighted UniFrac distances, comparing the bacterial communities among samples from cholelithiasis (red circles, *n* = 14) and control group (blue circles, *n* = 13). Percentages shown in the axes represent the proportion of dissimilarities. Analysis of molecular variance (AMOVA) was used to assess the statistical significance of the spatial separation between both groups (*p* value < 0.001)

The OTU-based microbial diversity was estimated. The intra-subject alpha diversity was estimated on data rarefied to the sequencing depth obtained in the sample with the lowest number of sequences (i.e., 20,000). The Shannon index (H) was determined for each sample. The median of this diversity index was statistically higher in the bile of the control group than that obtained in the patients with gallstones (Mann-Whitney *U* test, *p* value = 0.038) (Fig. 4). On the other hand, after applying Principal Coordinate Analysis (PCoA) to the weighted UniFrac distance matrix generated from the comparison between the microbiota identified in bile samples of cholelithiasis patients vs non-cholelithiasis (control group), we noticed that the non-cholelithiasis bile samples grouped together showing more similarities to each other, than to those samples from the cholelithiasis patients (Fig. 5). When analysis of molecular variance (AMOVA) was used to assess the statistical significance of the spatial separation observed, significant differences in the microbial clustering between both groups were revealed (*p* value < 0.001).

### Bile metabolomics

Neither the pH values nor the UV/visible spectra showed statistical differences between bile samples of both groups (data not shown). A pH mean value of 7.51 ± 0.88 was obtained for the bile samples. In relation to the absorbance spectra, two samples from the cholelithiasis group (C-09 and C-10) showed a blood-stained aspect, showing a maximum in 415 nm. This was not observed in any of the bile samples from the control group.

**Fig. 6:**
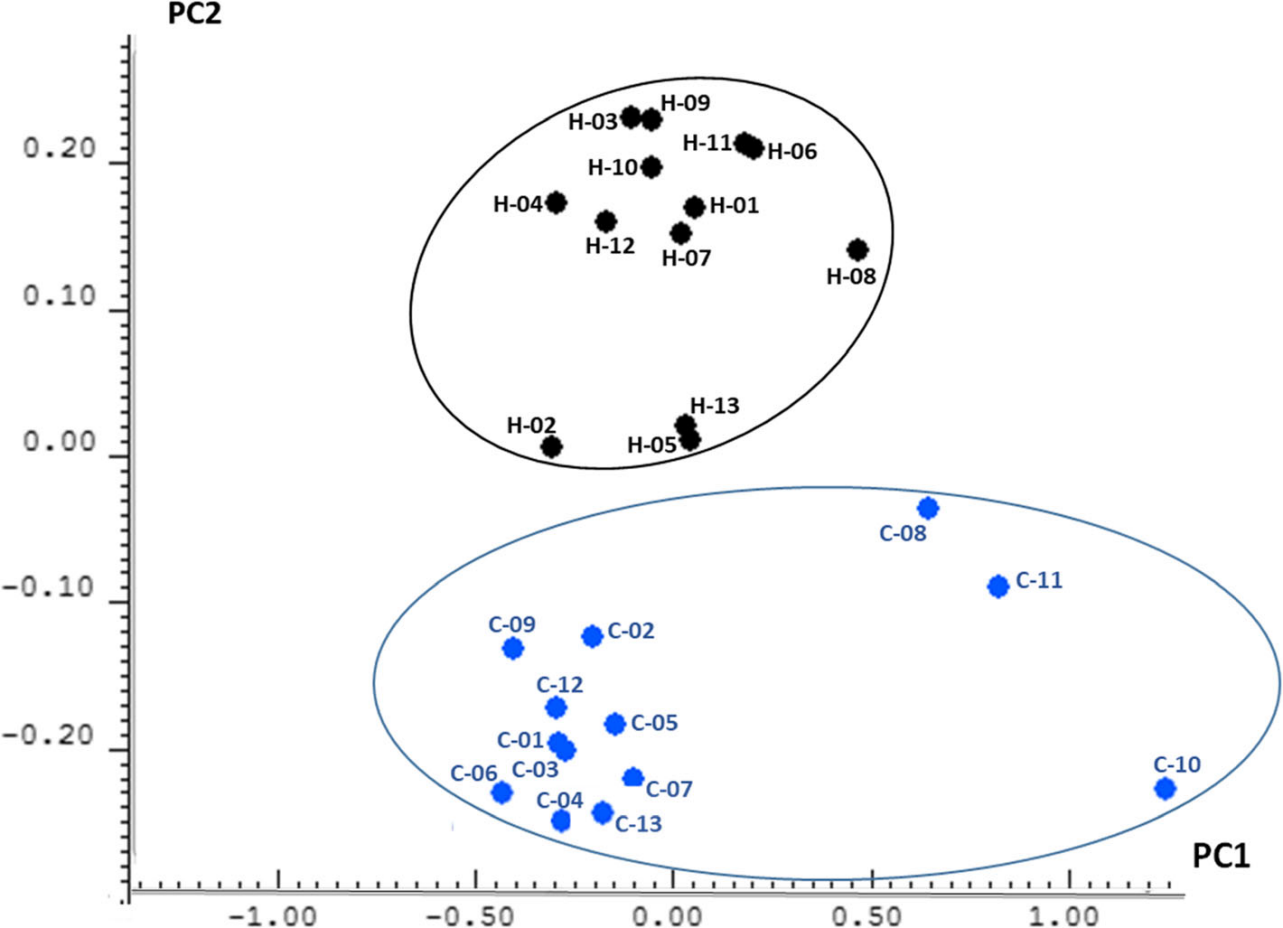
Principal component analysis (PCA) plot of bile metabolites. PC1 versus PC2 obtained for the whole spectra is represented. Control samples are represented in black. Blue dots belong to bile samples from cholelithiasis patients

The assignment of the signals from ^1^H-NMR spectrum is depicted in Table 6. Signals from glycine/taurine-conjugated BA, phosphatidylcholine, unspecific methylene groups from glycerides, cholesterol, and primary BA characterized the spectra of the samples of gallbladder bile. Considering the signals of the whole spectra, a statistically significant separation between bile samples of the two groups was detected (*p* value < 0.05) by principal component analysis (PCA) (Fig. 6). Besides, the split of the spectrum in two sections (0.15–4.20, aliphatic; 5.00–10.00, aromatic) led to a significant discrimination of the samples depending on types of individuals (patients vs controls). Therefore, the differences in the aliphatic section could be related to the content of chenodeoxycholic (CDCA), deoxycholic acid (DCA), and cholic acids (CA), whereas the differences within the aromatic section would be caused by their glycine- and/or taurine-conjugated forms. The highest percentage of explained variance was obtained by considering the whole spectrum, thus explaining a total of 76.23% in a plot of PC1 (63.06 %) vs PC2 (13.17 %). PCA results evidenced that the second principal component was closely related to bile sample type (controls “H” vs cholelithiasis “C”). It was observed that the control samples showed positive values for PC2, whereas those from patients with cholelithiasis presented negative values (Fig. 6).

**Table 6:** Signal assignments for ^1^H-NMR spectra obtained from gallbladder bile

Chemical shift	Compounds
0.00	TSP (internal standard)
0.67	BA (H18)
0.71	Cholesterol (H18)
1.42	Lipids (CH2)_n_
1.49	BA cholesterol + lipids
2.38	BA cholesterol + lipids
2.74	Lipids
2.78	Lipids
2.81	Lipids
3.07	Conjugated taurine
3.24	Phosphatidylcholine
3.49	CA+CDCA
3.56	DCA + conjugated taurine
3.63	Phosphatidylcholine
3.74	Conjugated glycine
3.84	CA+DCA
3.88	CA, CDA, + PC-glycerol
4.28	PC-glycerol
4.31	Phosphatidylcholine
4.44	PC-glycerol
4.46	PC-glycerol
4.83	Residual water
5.32	Lipids, cholesterol PC-glycerol
7.83	Glycochenodeoxycholic acid
7.86	Glycodeoxycholic acid
7.88	Glycocholic acid
7.99	Taurochenodeoxycholic acid
8.00	Taurodeoxycholic acid
8.01	Taurocholic acid

*BA* bile acids, *CA* cholic acid (primary), *CDCA* chenodeoxycholic acid (primary), *DCA* deoxycholic acid (secondary), *TSP* 3-(trimethylsilyl) propionicacid-d4, *PC-glycerol* phosphatidylcholine-glycerol

## Discussion

The advent and refinement of novel sequencing methodologies during the 21st century, the so called next-generation sequencing (NGS) methods, together with bioinformatics-based analyses, have revolutionized how we study the human microbiome. Thanks to these techniques, we are able to extract the information encrypted in the genomes of the intestinal microbiota members, as well as to depict the diversity and potential metabolic capabilities of this microbial community. The human fecal microbiota has largely been studied, and numerous links have been established between these microbial communities and different physiological conditions, a process that has been favored, at least in part, due to the ease of obtaining non-invasive biological samples. The characterization of other human microbial niches has been hampered by difficulties in accessing solid biopsies or liquid fluids and the lack of adequate methodologies to assess molecular studies in microbial ecosystems with a low bacterial load. Within these “other microbiotas,” recent evidence suggests that a variety of internal biological fluids possess a native microbiota, among which human milk and blood have received particular attention [7, 8, 21, 22]. However, caution should be taken for the presence of contaminating DNA when applying sequence-based techniques to the study of the microbiome in low biomass environments [23]. In our study, we analyzed the microbial load of human bile by qPCR. Remarkably, our quantitative results might be underestimated as we observed that, after enrichment of bile samples with different amounts of *L. lactis* cells, the bacterial cells quantified by 16S rRNA gene analysis were usually lower than the total number of cells added to the bile sample, suggesting that the primer choice or the cell lysis method could influence quantitative estimations.

To date, the microbiota of the human gallbladder has scarcely been studied, and current results are limited to a few hepatobiliary pathological conditions in which the gallbladder has been resected during surgery. Cultivation and culture-independent techniques have shown that enterobacteria are frequently isolated and detected in cholecystitis and cholelithiasis samples, although the lack of optimal culture conditions suitable for other microorganisms could allow those adapted to the biliary niche to pass unnoticed [11–13]. Remarkably, besides some fecal bacterial indicators of cholelithiasis, such as the genera *Roseburia* and *Oscillospora* [24], one of the few bacteria frequently associated with the presence of gallstones is *Salmonella enterica*, which is able to colonize and persist in the human gallbladder [25]. Only recently, using massive sequencing techniques, a few reports analyzed the biliary microbiome of the human gallbladder potentially associated with the generation of gallstones; however, the relatively low number of sequences analyzed and the absence of gallbladder control samples (samples from individuals without hepatobiliary pathology) hampered the yield of physiologically relevant results and made challenging to establish a microbiota profile associated with cholelithiasis. Among these studies, the analysis of a group of 29 Chinese patients with gallbladder gallstones showed that the biliary core microbiome was constituted by 6 phyla: *Actinobacteria*, *Bacteroidetes*, *Firmicutes*, and *Proteobacteria* being the predominating phyla, and *Bacteroides* the most abundant genus [17]. Similar results were found by Saltykova and co-workers, who observed that these four phyla dominated the human gallbladder microbiota of patients with gallbladder gallstones [18]. Our results also point to the predominance of these four phyla in bile obtained from the gallbladders of cholelithiasis patients, all of them with gallbladder gallstones, and the highest number of sequences assigned to a specific genus was for *Bacteroides* (Table 5). On the other hand, a few works have analyzed the human microbiota of samples from the common bile duct, using endoscopic retrograde cholangiopancreatography (ERCP) for sample collection, obtained from patients with gallstones in the common bile duct [14–16]. Overall, these studies showed a high abundance of the phyla *Firmicutes*, *Bacteroidetes*, and *Proteobacteria*, with a lower representation of *Actinobacteria* and other phyla.

Until now, the microbial populations inhabiting the human gallbladder in individuals without any hepatobiliary disorder had not been studied. Our group has recently shown that healthy pigs have a native microbiota [19], and this led us to think that humans, even in the absence of pathologies, can also hold a native gallbladder microbiota. Thus, in our study, we have tried to take another step in the characterization of the human bile microbiome, and in order to compare the microbial profile of cholelithiasis patient’s vs a non-pathological condition, we have established a control or reference group with samples from individuals without any hepatobiliary disease. Given the difficulty of obtaining bile samples from healthy individuals due to obvious ethical reasons, we have tried to overcome the problem to obtain samples from a “healthy group” by collecting samples from liver donors without any record of biliary or hepatic disorders. Since antibiotic prophylaxis is a standard protocol for liver transplantation, with the aim of minimizing the potential effect of antibiotics on the biliary microbiota, we only selected samples from liver donors that had received antibiotics at the intensive care unit (ICU) for less than 24 h before the surgery. Even so, we are aware that this antibiotic treatment (see the “Methods” section for details) may constitute a limitation of our study and could affect the endogenous microbiota of bile. Regrettably, as commented above, this was due to the difficulty in obtaining bile samples from healthy volunteers. In the bile samples of this control group, we found sequences matching some genera of the Alpha division of *Proteobacteria* (*Bradyrhizobium*, *Methylobacterium*, and *Sphingomonas*) that were previously associated with nitrogen fixation and potential contaminants of ultrapure water systems [26]. In this regard, with our template-free “blanks” (in which the bile sample had been substituted by molecular biology grade water in the DNA extraction procedure), no amplification from the 16S rRNA gene was achieved, suggesting an extremely low bacterial load in the water, and sequencing results could not be obtained. Therefore, we were not able to confirm if these genera (representing around 10% of total assigned reads) constitute a potential contamination or background signals, a fact that is plausible in high-throughput sequencing approaches using biological specimens with low bacterial load, as is the case of our control bile samples (Fig. 3). Therefore, we cannot completely rule out the possibility of introducing a minor bias in our sequence-based microbiome study of bile control samples from liver donors, but shotgun metagenomic analyses corroborated the data of 16S rRNA gene profiling, indicating the existence of three main phyla (*Actinobacteria*, *Bacteroidetes*, *Firmicutes*) apart from *Proteobacteria* (Table 2) in human bile samples, which is in accordance with previous literature of this environment [17, 18]. In addition, from the liver donors, we extracted DNA from gallbladder tissues removed during liver transplantation surgery. 16S rRNA gene profiling was carried out in three of these biopsies (from individuals H-04, H-05, and H-06), revealing the presence of a low abundance of bacterial amplicons, mainly belonging to *Firmicutes*, *Bacteroidetes*, and *Actinobacteria* phyla. Observed eukaryotic amplicons ranged between 75 and 85% of the total profile. Based on our previous experience, such results indicate that the vast majority of the retrieved DNA is eukaryotic (data not shown).

Furthermore, weighted PCoA analysis grouped the biliary microbiota of the two study groups (non-lithiasis vs lithiasis patients) differently, showing a higher dispersion among the samples from cholelithiasis patients. Statistical differences in the microbial relative abundances of sequences between both groups, at family and genus level, were also noticeable. Within the different genera detected, the highest statistical significance was observed for *Bacteroides* and *Escherichia-Shigella* (more abundant in the cholelithiasis patients) and *Sphingomonas* (more abundant in the control group). As previously mentioned, enterobacteria are frequent in the bile of cholelithiasis patients [11–13]. Furthermore, it is worth highlighting that half of the cholelithiasis patients have altered levels of total cholesterol or triglycerides at the time of sampling, or a clinical history of hypercholesterolemia and/or obesity (Table 1 and Additional file 1: Table S1). In this regard, high abundance percentages of *Bacteroides* in the fecal microbiota have been linked to the consumption of a diet rich in animal fat [27, 28]. In accordance with our microbiological and metabolic results found in bile from patients with cholelithiasis, it has been previously reported that high-fat intake increases BA secretion, and accordingly, animal-based diets increase the abundance of bile-tolerant microorganisms in the gut, such as *Bacteroides* [28]. Although results on human intestinal microbiota cannot be directly compared with the biliary microbiota described in our work, we hypothesized that diet rich in saturated fat and cholesterol levels, two factors that have been related to the appearance of cholelithiasis [29], might favor microorganisms, such as *Bacteroides*, in the biliary micro-environment.

From the 16S rRNA gene profile analysis, it seems that 3 to 4 phyla constitute the core population of human bile, and relevant differences at family and genera level are highlighted depending on the specific physiological condition of the host.

After a careful selection of samples from the control group, only three of them with a reasonably high 16S/18S ratio were used for shotgun metagenomic analysis. We have seen that the distribution of the main functional categories in the biliary metagenome is similar to that described in the human gut microbiota [30]. However, it is worth highlighting that genes related to the metabolism of cholesterol and BA biosynthesis are present at higher relative abundance in the human bile microbiome with respect to that of the human gut [20]. This could reflect the adaptation of the bile microorganisms to this specific niche. However, the interpretation of these results should be taken with caution, due to the small number of samples analyzed from the control group (3 individuals) and to the particular physiological conditions of the liver donors (a period of stay in the hospital’s ICU and the unavoidable standard-of-care antibiotic treatment), considering that the comparison of gallbladder and fecal microbiotas was not carried out within the same group of individuals (liver donors who had suffered a brain accident or stroke vs healthy individuals).

Regarding the biochemical analysis of the bile samples, the pH values of the samples were within the range previously described in bile from gallbladders [31]. Attending to the color analysis, the maximum at 415 nm of two samples coming from patients with cholelithiasis could be associated to hemoglobin [32]. Together, the differences in shapes of the spectra of the rest of samples, although not statistically significant, could be related to the heterogeneity on the concentration of pigments (bilirubin and biliverdin) and bile salts, the aggregation state, and the oscillations of pH values [33, 34].

With respect to the metabolic NMR profiles (Table 6), high levels of the secondary BA deoxycholic acid (DCA) in blood and feces have been associated with increased risk of cholesterol gallstone disease [35]. Statistically significant differences between bile samples from controls and patients were found when considering the compounds of both the aromatic and the aliphatic regions of the spectra (Fig. 6). In agreement with this, several authors have stated that different levels of BA or its glycine and/or taurine conjugates play an important role in cholestasis [36, 37]. Besides, Ijare and colleagues described a major role of the amide proton region of the ^1^H-NMR spectra of human bile in differentiating cholestatic patterns from normal ones [38]. Further studies are mandatory to characterize in depth the potential relationships between the biliary metabolic and microbial profiles.

## Conclusions

This work is the first study pointing to the existence of a human gallbladder microbiota in individuals without any hepatobiliary disease and establishing a link between bile microbiota and pathological conditions. The 16S rRNA gene profiling and the metagenomic analysis allowed us to propose the existence of a microbial bile ecosystem and to access the main taxonomic and functional profiles present in this microbiome. Furthermore, the metabolomics analysis showed that the two study groups have different metabolic profiles. We are aware of the moderate sample size of our work, mainly related to the number of liver transplants carried out in the collaborating hospitals during the one and a half year sampling period and to the strict inclusion criteria for the two study groups. Also, the difficulty of obtaining gallbladder samples from healthy volunteers and the inherent disadvantage of working with biological samples with a low bacterial load must be taken into account. In addition, we must bear in mind that the results of the control group have been obtained with liver donors that have been subjected to the specific protocols of the hospital’s ICU, including an unavoidable standard-of-care short antibiotic treatment that could introduce a bias in the physiologically normal biliary microbiota. Even considering these limitations, our results establish the basis for future larger-scale studies on the relationship between bile microbiota, gut microbiota, human metabolism, and health. Future investigations should be oriented to unraveling the role and influence of the biliary microbiota in the pathophysiology of this (lithiasis) and other human diseases related to diet, bile, and cholesterol metabolism. These findings might open promising strategies to search for novel biomarkers associated with the disease or dietary strategies that could help prevention and/or patient care and treatment.

## Methods

### Bile samples and patients

Human bile samples from the gallbladders of individuals without hepatobiliary disease were obtained during liver transplants from liver donors who had suffered a brain accident or stroke (control group or reference group; H-sample codes). Although a total of 26 donors were initially recruited and sampled, a further selection of bile samples for the microbiological study was applied in order to have a homogenous reference group. The criteria used for selection were as follows: less than 80 years old, no more than a 48-h stay in the hospital’s ICU before the transplant, and had not received antibiotics at ICU for more than 24 h. In all selected cases, the antimicrobial treatment consisted on 2 g of amoxicillin-clavulanic acid every 6 h until liver transplantation surgery. Other characteristics of this selected control group (*n* = 13) were as follows: 4 male and 9 female and aged range 37 to 79 years old. The samples of bile from the gallbladders were collected aseptically during the surgery by physicians at the General Surgery Service of HUCA (Central University Hospital of Asturias, Spain). The surgeons also confirmed the absence of gallstones and sludge from the donors.

Bile samples from gallbladders from a similar group in gender and age (3 male and 11 female, age range 27 to 73 years old), with a bile disorder (diagnosed with cholelithiasis), were obtained from 14 patients (*n* = 14) with gallstones, who underwent surgical gallbladder removal at Cabueñes Gijon University Hospital (Asturias, Spain) (Cholelithiasis group; C-sample codes). General characteristics and clinical parameters of the patients are reported in Table 1. None of the patients received previous antibiotic prophylaxis (which was considered as an exclusion criterion), and the intervention was performed in all of them by laparoscopic cholecystectomy. The recruitment of patients was undertaken by physicians of the General and Digestive Surgery Service of Cabueñes Gijon University Hospital, Spain. After bile collection, bile samples were immediately transported refrigerated to the laboratory and stored at − 80 °C until use. Comparison between clinical parameters of both groups of individuals (patients with cholelithiasis and liver donors as controls), from whom bile samples were obtained in this study, is reported in Additional file 1: Table S1. Additionally, there were no statistical differences with respect to gender (79% female and 21% male in patients with cholelithiasis vs 69% female and 31% male in controls) and age (52 years old in patients vs 59 in controls) between both groups.

Ethical approval for this study was obtained from the Bioethics Committee of CSIC (Consejo Superior de Investigaciones Científicas) and from the Regional Ethics Committee for Clinical Research (Servicio de Salud del Principado de Asturias n°112/13) in compliance with the Declaration of Helsinki of 1964. All experiments were carried out in accordance with the approved guidelines and regulations.

### Microbial analysis of bile samples

#### DNA extraction and quantification

Total DNA extraction from 1-ml bile samples was performed following an optimized protocol based on a previously described method [19], with slight modifications. Briefly, samples were centrifuged at maximum speed at room temperature for 10 min and pellets re-suspended in 0.5 ml of extraction buffer consisting of 200 mM Tris-HCl pH 7.0, 25 mM EDTA, 250 mM NaCl, and the following enzymes: 20 mg/ml lysozyme (Merck, Darmstadt, Germany), 5 μg/ml of lysostaphin (Sigma-Aldrich, Saint Louis, MO, USA), and 40 U/ml mutanolysin (Sigma-Aldrich). Enzymatic lysis was performed for 1 h at 37 °C; after that, SDS was added to a final concentration of 0.5% (*w*/*v*), and mechanical disruption was performed in a FastPrep FP120 apparatus (Qbiogene, Carlsbad, CA, USA). The lysate solution was treated with proteinase K, 1.5 M NaCl as previously described [39] and extracted with phenol/chloroform. Precipitation of DNA was performed with 0.1 volumes of 3 M sodium acetate pH 5.2 and 2.5 volumes of cold ethanol. The DNA was then pelleted, washed with 70% ethanol, resuspended in 50 μl of molecular-biology grade water (Sigma-Aldrich), and stored at − 20 °C until use. DNA concentration and quality was determined in a BioTek Epoch™ spectrophotometer system (Thermo Fisher Scientific, Inc., Waltham, MA, USA) and in parallel in a Qubit fluorometer with dsDNA assay kits (Thermo Fisher Scientific).

#### Determination of prokaryotic and eukaryotic DNA by qPCR

Differentiation and quantification of eukaryotic and prokaryotic DNA from bile samples were carried out by qPCR using specific primers targeting the 18S rRNA gene of eukaryotic cells [40] and the 16S rRNA gene of prokaryotic microorganisms [41]. Amplification reactions were performed in 96-well optical plates (Applied Biosystems, Foster City, CA, USA) in a 7500 Fast RealTime PCR System (Applied Biosystems). Amplifications were done in triplicate in a final volume of 25 μl containing 2× SYBR Green PCR Master Mix (Applied Biosystems), 0.2 μM of each primer, and 1 μl of DNA obtained from bile. Primer efficiency was calculated from the slope of the standard curve (*E* = 10^−1/slope^). Standard curves were generated by plotting the Ct values against the numbers of cells corresponding to serial tenfold dilutions of cultures of *L. lactis* strain NZ9000 as a reference for prokaryotic DNA and calculated by plate counting [42] and HT-29 cell line as a reference for eukaryotic DNA and titrated under an inverted microscope with a Neubauer Chamber [43]. To simulate different bacterial loads in the bile matrix, human bile was artificially supplemented with serial tenfold dilutions of known concentrations (ranged from 10^2^ to 10^7^ cfu/ml) of a grown culture of *L. lactis* strain NZ9000.

#### Shotgun metagenomic sequencing and analysis

Extracted DNA of three bile samples from the control group were used for total shotgun sequencing. DNA was fragmented to 550–650 bp using a BioRuptor machine (Diagenodo, Belgium) and subsequently prepared with the Next era XT Library Preparation kit (Illumina, San Diego, CA, USA). Sequencing was performed at the DNA sequencing facility of GenProbio SRL (Parma, Italy) using an Illumina MiSeq System with MiSeq Reagent Kit v3 chemicals (Illumina).

The fastq files were filtered for reads with quality < 25 and presence of alien DNA, as well as for reads < 150 bp. Bases were also removed from the end of the reads until the average quality in a window of 5 bp was > 25. Only paired data was further analyzed. Assembly of the shotgun dataset and annotation of the reads was performed with the MEGAnnotator platform [44]. Taxonomic classification was obtained using a combination of RapSearch2 software [45] and the non-redundant NCBI database (BLASTx), and MEGAN5, in order to reconstruct taxonomic profiles based on reads covering coding regions. Functional analysis of the reads was achieved using CAZy [46], EggNOG [47], and MetaCyc [48] databases. Specifically, COG functional class profiling was based on the EggNOG database; meanwhile, metabolic pathway prediction was based on the MetaCyc database. Abundance of genes related to the metabolism of cholesterol and BA was compared with those found in fecal metagenomes of five healthy individuals used as controls in a previous study [20]. These dataset sequences are available at the Sequence Read Archive (SRA) database under the accession number SRP125191.

#### High-throughput sequencing and analysis of 16S rRNA gene amplicons

Partial 16S rRNA gene sequences were amplified from the extracted DNA of 27 bile samples (13 controls and 14 patients) using the primer’s pair Probio_Uni and /Probio_Rev, which target the variable region V3 of the bacterial 16S rRNA gene, as previously described [49]. Samples were submitted to 2 × 250 bp paired-end sequencing by means of an Illumina MiSeq System (Illumina). Sequence reads were filtered by the Illumina software to remove low-quality sequences. All Illumina quality-approved, trimmed, and filtered sequences were processed using a custom script based on the QIIME software suite [50]. After joining the paired-end reads, the quality control phase retained sequences with a mean sequence quality score > 20 and a length between 140 and 400 bp. Sequences with homopolymer regions > 7 bp and those with mismatched primers were omitted. In order to calculate downstream diversity measures, 16S rRNA Operational Taxonomic Units (OTUs) were defined at 100% sequence homology using DADA [51]; OTUs not encompassing at least 2 sequences of the same sample were removed. All sequences were classified to the lowest possible taxonomic rank using QIIME and the SILVA database as reference [50, 52]. Similarity of the bacterial communities between samples was calculated by weighted UniFrac method [53]. All raw data was deposited in the SRA of the NCBI (https://www.ncbi.nlm.nih.gov/sra) under accession numbers SRR6872870 to SRR6872896 (for the 16S rRNA gene sequencing data) and SRR6877525 to SRR6877527 (for the shotgun sequencing data).

### Metabolic analysis of bile samples

#### pH and color determination

pH and UV-visible spectra were measured on each of the collected bile samples. A CRISON GLP21 pH-meter was used to the pH measurement. Optical density was measured between 200 and 800 nm in a spectrophotometer Zenyth 200RT (Anthos Labtec Instruments GmbH, Salzburg, Austria).

#### Sample preparation for NMR analysis of metabolites

The same samples used for the microbiota analysis were used for the NMR analysis, excluding sample C-14 (this sample was not available after the 16S rRNA gene profiling and the optimization of analyses protocols). The intact bile samples were frozen at − 80 °C, freeze-dried, and stored under vacuum at − 80 °C. For NMR analyses, 5 mg of bile and 50 μl of a 1 mM solution in distilled water of the sodium salt of the 3-(trimethylsilyl) propionicacid-d4 (TSP) were mixed with 550 μl of distilled water, and the mix was transferred to 5-mm diameter NMR glass tubes. TSP was added to provide an internal reference standard and a field-frequency lock (0.00 ppm).

#### 1D ^1^H-NMR experiments

NMR experiments were performed on a Bruker Biospin Avance 700-MHz NMR spectrometer. Spectra were recorded using the following pulse sequences: 1D NOESY (NOESYPRESAT) and CPMG (Carr Purcell Meiboom Gill), both with water suppression. The 1D NMR experiments were acquired with 32k time domain data points, 18 ppm spectral width, 64 scans, and 2 s of relaxation delay. A mixing time of 150 ms was used in the 1D NOESY experiment and an echo time of 50 ms was selected for the CPMG sequence. The free induction decays (FIDs) obtained were processed with Bruker BioSpin TOPSPIN software (version 3.1) as follows: exponential filtering (LB ¼ 1 Hz), Fourier transformation (FT), spectral phasing, and baseline correction.

#### 2D ^1^H-NMR experiments

Assignments of NMR signals were based on standard two dimensional experiments, namely, ^1^H-^1^H COSY, ^1^H-^1^H TOCSY, ^1^H-^13^C HSQC, and ^1^H-^13^C HMBC. Homonuclear water suppressed COSY and TOCSY experiments were performed with 2k data points in t_2_ domain and 384 increments in t_1_, each with 64 scans. Spectral widths of 13 ppm were employed in both dimensions. Mixing times of 70 ms were used for TOCSY experiments. Heteronuclear HSQC and HMBC experiments of these liquid samples were performed as follows: 2k data points in t_2_ domain and 384 increments in t_1_, each with 256 scans; spectral widths of 13 ppm for f_2_ dimension and 260 ppm for f_1_ dimension; coupling constant values of 145 Hz and 10 Hz were employed to set delay durations for short range and long range correlations, respectively. In all cases, two-dimensional data sets were enhanced in the first dimension by forward linear prediction from 384 to 512 real data points followed by zero filling to 1024 data points. In addition, squared sine bell window functions were applied in both dimensions prior to Fourier transformation. Assignment of resonances in the ^1^H-NMR spectra was based on both spin connectivity information obtained from 2D experiments and the use, as guidelines, of both data reported in the literature [36] and data obtained from HMDB database (Human Metabolite Data Base http://www.hmdb.ca/).

### Statistical analyses

The identification of differentially abundant taxa was assessed using the Metastats program [54]. Multiple hypothesis tests were adjusted using the false discovery rate (FDR) correction; an FDR threshold of 0.25 was used to identify significant differences. Multivariable statistical analysis of microbiological sequences was performed by PCoA. Differences in the microbial distribution were sought by AMOVA. Comparisons among other variables were performed by using the non-parametric Mann-Whitney *U* test. A PCA was developed on NMR signals as discrimination analysis. Statistical analysis was performed using SPSS v. 22.00 (IBM, Armonk, NY, USA) and the free software R (www.r-project.org).

## Additional file

Additional file 1: **Table S1.** Data and clinical characteristics of both groups of individuals (patients with cholelithiasis and liver donors as controls) from whom bile samples were analyzed in this study. (DOCX 19 kb)

## Data Availability

All raw data was deposited in the Sequence Read Archive (SRA) of the NCBI (https://www.ncbi.nlm.nih.gov/sra) under accession numbers SRR6872870 to SRR6872896 (for the 16S rRNA gene sequencing data) and SRR6877525 to SRR6877527 (for the shotgun sequencing data).
