# Supplementary material for: The human gallbladder microbiome is related to the physiological state and the biliary metabolic profile
Source: Microbiome. 2019 Jul 4;7:100. doi: 10.1186/s40168-019-0712-8 (PMC6610825; doi:10.1186/s40168-019-0712-8)
Supplement: Supplementary file 1 — Table S1. Data and clinical characteristics of both groups of individuals (patients with cholelithiasis and liver donors as controls) from whom bile samples were analyzed in this study. (DOCX 19 kb) [file 40168_2019_712_MOESM1_ESM.docx]

**Table S1.** Data and clinical characteristics of both groups of individuals (patients with cholelithiasis and liver donors as controls) from whom bile samples were analyzed in this study.

| Parameter | Cholelithiasis^a^ (n=14) | Liver donors (n=13) | *p*-value^b^ |  |
| --- | --- | --- | --- | --- |
| Age | 52±14 | 59±14 | 0.274 |  |
| Triglycerides (50-165mg/dl)^c^ | | 113±82.69 | 76.73±38.60 | **0.044** |
| Total colesterol (125-240mg/dl) | | 192.79±53.32 | 131.64±45.46 | **0.011** |
| HDL (40-80mg/dl) | | 49.93±14.25 | 44.27±17.68 | 0.434 |
| LDL (0-160mg/dl) | | 117.07±36.40 | 72.18±35.48 | **0.011** |
| Glucose (65-100mg/dl) | | 79.07±16.95 | 123.91±38.16 | **0.000** |
| Urea (10-60mg/dl) | | 26.80±8.02 | 36.00±16.53 | 0.197 |
| Creatinine (0.55-1.2mg/dl) | | 0.66±0.19 | 1.01±0.53 | 0.150 |
| ALT (4-41U/l) | | 27.64±13.11 | 53.45±83.24 | 0.851 |
| AST (4-35U/l) | | 28.28±10.26 | 75.5±126.91 | 0.767 |
| GGT (1-24U/l) | | 35.64±28.54 | 44.63±41.57 | 0.809 |
| Alkaline phosphatase (40-117U/l) | 81.85±42.26 | 68.36±16.45 | 0.791 |  |

^a^ Mean ± standard deviation.  ^b^ Comparisons were made with the statistical non-parametric. Mann-Whitney U test and significance (in bold) was considered with a *p*-value below 0.05. ^c^ Normal ranges in parenthesis

Abbreviations: HDL. Cholesterol HDL; LDL. Cholesterol LDL; GGT. Gamma-glutamyl transferase; ALT. Alanine aminotransferase; AST. Aspartate aminotransferase.
